# Design of a Phase 3, Multicenter, Randomized, Placebo-Controlled, Double-Blind Study of Nipocalimab in Pregnancies at Risk for Fetal and Neonatal Alloimmune Thrombocytopenia

**DOI:** 10.1055/a-2666-5642

**Published:** 2025-08-20

**Authors:** Heidi Tiller, Eleonor Tiblad, Pamela Baker, Hillary Van Valkenburgh, Dirk Heerwegh, Babajide Keshinro

**Affiliations:** 1 Department of Clinical Medicine, UiT The Arctic University of Norway, Tromsø, Norway; 2 Department of Obstetrics and Gynecology, University Hospital of North Norway, Tromsø, Norway; 3 Clinical Epidemiology Division, Department of Medicine, Solna, Karolinska Institutet, Stockholm, Sweden; 4 Johnson & Johnson, Spring House, Pennsylvania; 5 Johnson & Johnson, Beerse, Belgium; 6 Johnson & Johnson, Biologics BV, Leiden, The Netherlands

**Keywords:** FNAIT, human platelet antigen, FcRn, nipocalimab, safety, efficacy, study design

## Abstract

**Objective:**

Nipocalimab, a neonatal Fc receptor blocker, inhibits transplacental transfer of maternal immunoglobulin G (IgG) and lowers circulating maternal IgG levels. In a phase 2 study, nipocalimab demonstrated evidence of safety and efficacy in delaying or preventing fetal anemia in early-onset severe hemolytic disease of the fetus and newborn, suggesting a potential benefit in other IgG alloantibody-mediated perinatal diseases, including fetal and neonatal alloimmune thrombocytopenia (FNAIT). The phase 3 FREESIA-1 study aims to evaluate the safety and efficacy of nipocalimab in at-risk FNAIT pregnancies.

**Study Design:**

This multicenter, placebo-controlled, double-blind, phase 3 study will enroll human platelet antigen (HPA)-1a-alloimmunized pregnant individuals with an HPA-1a-positive fetus and prior FNAIT-affected pregnancy without intracranial hemorrhage or severe bleeding in the fetus/newborn. Participants will be randomized 2:1 to weekly intravenous nipocalimab or placebo at 13 to 18 weeks of gestation until delivery. Maternal participants will receive ultrasound monitoring approximately every 2 weeks during treatment. Neonates will receive a cranial ultrasound scan, platelet count assessment, and, if needed, platelet transfusion. Maternal participants will be followed for 24 weeks and neonates/infants for 104 weeks.

**Results:**

The primary endpoint is an adverse outcome of fetal death or adjudicated severe bleeding in utero up to 1 week postbirth, or neonatal platelet count at birth < 30 × 10 ^9^ /L. Key secondary endpoints include adjudicated bleeding in utero up to the first week postbirth in fetuses/neonates and platelet count at birth in neonates. Additional secondary endpoints in fetuses/neonates include death; platelet count at birth <10, <30, <50, and <150 × 10 ^9^ /L; nadir platelet count over the first week postbirth; platelet transfusion; adjudicated severe bleeding up to the first week postbirth; and postnatal intravenous immunoglobulin for thrombocytopenia. Other assessments include safety, patient/caregiver-reported outcomes, pharmacokinetics, pharmacodynamics, and immunogenicity of nipocalimab.

**Conclusion:**

FREESIA-1 is the first placebo-controlled, randomized, multicenter trial designed to evaluate the safety and efficacy of nipocalimab in at-risk FNAIT pregnancies. (ClinicalTrials.gov Identifier: NCT06449651. Accessed at: https://clinicaltrials.gov/study/NCT06449651 . Date of registration: June 10, 2024.)

**Key Points:**

- FNAIT can lead to fetal/neonatal mortality and morbidity.
- Nipocalimab blocks IgG recycling and placental transfer.
- Nipocalimab may reduce adverse outcomes of FNAIT.
- FREESIA-1 will evaluate nipocalimab in FNAIT.

Fetal and neonatal alloimmune thrombocytopenia (FNAIT) is a rare pregnancy complication reported to occur in approximately one per 1,000 to 2,000 pregnancies. 1 2 FNAIT occurs when there is incompatibility between human platelet antigen (HPA) types between a pregnant individual and their fetus. 2 3 Maternal immunoglobulin G (IgG) alloantibodies against fetal HPA antibodies cross the placenta through the neonatal Fc receptor (FcRn), 4 resulting in the destruction of platelets and causing various degrees of thrombocytopenia in the fetus and neonate. 2 Maternal alloimmunization against HPA-1a is the most common cause of FNAIT, occurring in 80 to 85% of FNAIT cases. 2 5 6

In the most severe FNAIT forms, fetal/neonatal intracranial hemorrhage (ICH) can occur, with an incidence of one per 10,000 pregnancies. 1 2 ICH is associated with fetal/neonatal death (35–48%) and neurological sequelae (53–70%) in most cases. 2 7 In pregnancies with a prior history of FNAIT without ICH or severe fetal/neonatal hemorrhage (defined as a standard risk group), the incidence of ICH is much lower compared with pregnancies having a prior history of FNAIT with ICH (defined as a high risk group). 8 9 10 High levels of maternal anti-HPA-1a antibodies were also found to be associated with placental chronic inflammation, placenta-associated biomarkers, and increased risk of lower birth weight. 11 12 13 14 15

There are currently no evidence-based preventive treatments for pregnancies in either standard-risk or high-risk groups. Off-label use of intravenous immunoglobulin (IVIG), with or without corticosteroids, has been associated with a significantly lower risk of ICH when used for pregnancies in the high-risk group. 3 7 16 However, the benefit of antenatal IVIG in standard-risk pregnancies remains questionable. 17 Despite its widespread off-label use, there is a lack of consensus regarding IVIG treatment regimens, with or without corticosteroids, in FNAIT management. 10 17 Of note, both IVIG and corticosteroids are associated with side effects and unclear risk-benefit profiles. 2 3 10 17 18 19 IVIG use is further limited by a high burden on patients, including global shortages and substantial investments in costs and resources. 17

Nipocalimab is a fully human, IgG1, high-affinity, aglycosylated, FcRn-blocking monoclonal antibody that blocks placental IgG transfer and lowers circulating maternal IgG levels available for transfer ( Fig. 1 ). 20 21 In an open-label, single-arm, phase 2 study of an analogous condition, early-onset severe hemolytic disease of the fetus and newborn (HDFN; ClinicalTrials.gov Identifier: NCT03842189), 20 nipocalimab demonstrated evidence for safety and efficacy in delaying or preventing fetal anemia that supports further investigation in an ongoing phase 3 trial of severe HDFN (ClinicalTrials.gov Identifier: NCT05912517). 22 These data provide support for studying nipocalimab for the treatment of other IgG alloantibody-mediated perinatal diseases, including FNAIT.

**Fig. 1:**
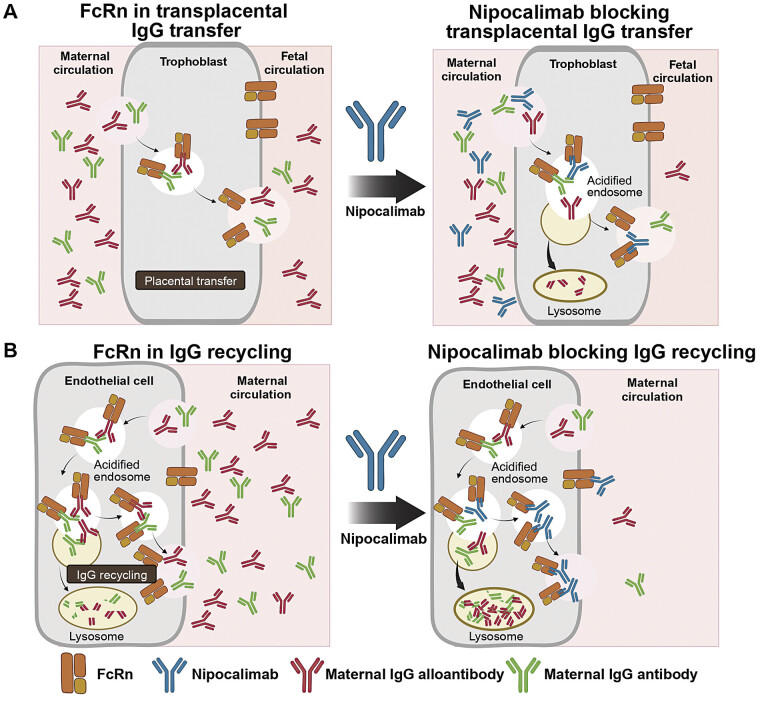
Anticipated prevention of FNAIT by nipocalimab through ( **A** ) blocking transplacental transfer and ( **B** ) IgG recycling in maternal circulation. 20 FcRn, neonatal Fc receptor; FNAIT, fetal and neonatal alloimmune thrombocytopenia; IgG, immunoglobulin G. Reproduced with permission from Massachusetts Medical Society. 20

Here, we present the design of FREESIA-1 (ClinicalTrials.gov Identifier: NCT06449651), a multicenter, randomized, placebo-controlled, double-blind, phase 3 study, which will evaluate the safety and efficacy of nipocalimab in pregnancies affected by FNAIT in the standard risk group.

## Materials and Methods

### Ethics

The FREESIA-1 trial is being conducted in compliance with International Council for Harmonisation guidelines on Good Clinical Practice 23 and applicable regulatory and local requirements. Approvals from an independent ethics committee and institutional review board are obtained for each participating center according to applicable national regulations.

All participants are fully informed of the risks and requirements of the study and receive any new information that may affect their decision to continue participation during the study. They are informed that their consent to participate in the study is voluntary and may be withdrawn at any time with no reason given and without penalty or loss of benefits to which they would otherwise be entitled. Only participants who are fully able to understand the risks, benefits, and potential adverse events (AEs) of the study, and provide their consent voluntarily, are enrolled.

### Participants

Eligible participants are 18 to 45 years of age, are pregnant at an estimated gestational age (GA) of 13 to 18 weeks at randomization based on ultrasound dating, have a history of FNAIT without fetal/neonatal ICH or severe hemorrhage in ≥1 prior pregnancy, and have the presence of maternal anti-HPA-1a alloantibodies and a positive fetal HPA-1a genotype in the current pregnancy (see complete inclusion criteria in Table 1 ).

**Table 1:** Complete inclusion and exclusion criteria

Inclusion criteria
• Female and 18 (or the legal age of consent if > 18 y in the local region) to 45 y of age at the time of informed consent • Pregnant and an estimated GA (based on ultrasound dating) from weeks 13 to 18 at randomization • Has a history of ≥1 prior pregnancy with FNAIT (including neonatal platelet count < 150 × 10 ^9^ /L), with none of them affected by fetal/neonatal ICH or severe hemorrhage based on medical records • Current pregnancy with presence of maternal anti-HPA-1a alloantibody and positive fetal HPA-1a genotype • Health status considered stable by the investigator on the basis of physical examination, medical history, vital signs, 12-lead ECG, and clinical laboratory tests performed at screening • Must sign an ICF indicating that the maternal participant understands the purpose of and procedures required for the study and is willing to participate in the study up to 24 wk of follow-up. The parents(s)/guardian(s) of the neonate/infant must also sign an ICF (as per local requirements) to permit 104-wk follow-up for the neonate/infant and agree to complete caregiver-reported outcomes for the infant • For the maternal participant and neonate/infant, willing to forego participation in another clinical study of an investigational therapy until the last follow-up visit • Must agree not to donate blood through the final follow-up visit at week 24 postpartum
Exclusion criteria
• Currently pregnant with multiple gestations (twins or more) • History of severe preeclampsia in a previous pregnancy • History of severe fetal growth restriction (birth weight < third percentile for GA) in a previous pregnancy • History of myocardial infarction, unstable ischemic heart disease, or stroke • Known allergies, hypersensitivity, or intolerance to nipocalimab or its excipients • Has a history of severe, progressive, and/or uncontrolled hepatic (e.g., viral/alcoholic/autoimmune hepatitis and/or metabolic liver disease), gastrointestinal, renal, pulmonary, cardiovascular, psychiatric, neurological or musculoskeletal disorder, hypertension, and/or any other medical or uncontrolled autoimmune disorder(s) (e.g., diabetes mellitus) or clinically significant abnormalities in screening laboratory that might interfere with the participant's full participation in the study or confound the protocol-specified assessments, and/or might jeopardize the safety of the participant, their fetus, or the validity of the study results • Has any confirmed or suspected clinical immunodeficiency syndrome or has a family history of congenital or hereditary immunodeficiency unless confirmed absent in the participant • History of solid organ or bone marrow transplantation (except for a corneal transplant performed ≥12 wk before screening) • Currently has a malignancy or has a history of malignancy within 3 y before screening (except for localized basal cell carcinoma and/or squamous cell skin cancer that has been adequately treated with no evidence of recurrence for ≥ 12 wk before the first study intervention administration or cervical carcinoma in situ that has been treated with no evidence of recurrence for ≥ 12 wk before the first study intervention administration) • Has shown a previous severe immediate hypersensitivity reaction such as anaphylaxis to therapeutic proteins (e.g., monoclonal antibodies) • History of serious infection that required hospitalization or parenteral antibiotics within 8 wk of screening or a history of recurring severe infections • Has a severe infection including opportunistic infections (e.g., pneumonia, biliary tract infection, diverticulitis, *Clostridium difficile* infection, cytomegalovirus, and pneumocystosis, aspergillosis) requiring parenteral anti-infectives or hospitalization, or is assessed as serious/clinically significant by the investigator, within 8 wk prior to screening • Has a severe chronic infection (e.g., bronchiectasis, chronic osteomyelitis, and chronic pyelonephritis) or requires chronic treatment with anti-infectives (e.g., antibiotics and antivirals) • Has tested positive for or been exposed to COVID-19 within 4 wk prior to the first dose of study intervention. Participants who have tested positive for or been exposed to COVID-19 may participate if they have both an absence of symptoms and a negative validated COVID-19 test obtained ≥ 2 wk after symptom onset (or the first positive test for asymptomatic infection) or exposure • Has received rituximab, eculizumab, or FcRn antagonists (e.g., efgartigimod) within 26 wk prior to screening • Is currently receiving systemic corticosteroids or other immunosuppressants • Use of low-potency topical corticosteroids, nasal/inhaled corticosteroids, or intraarticular corticosteroids is permitted • Has received or is planning to receive IVIG, plasmapheresis, immunoadsorption therapy, or any IgG Fc-related therapeutics during the current pregnancy • Has received a live virus vaccination during the current pregnancy or has a known need to receive a live virus vaccination during the study while receiving study intervention or within ≥ 8 wk after the last administration of study intervention in this study • Has received a BCG vaccination within 1 y prior to the first dose of study intervention or has a known need to receive the BCG vaccine during the study or within ≥ 8 wk after the last administration of study intervention • Has previously received nipocalimab or was enrolled and received study intervention in this study in a previous pregnancy • Is currently enrolled or plans to enroll in an investigational study and receive investigational intervention during the study • Has received an investigational intervention within the period ≤5 half-lives of the investigational compound prior to screening or used an invasive investigational medical device within 12 wk prior to screening • Has positive laboratory test results for HBV infection • History of any positive test for HIV at screening • Has an active infection at screening or baseline with Coxsackie, syphilis, cytomegalovirus, toxoplasmosis, parvovirus, or herpes simplex 1 or 2, as evidenced by clinical signs and symptoms and/or screening serology results from the central laboratory. Serology evidence of prior infection or exposure, but without clinical signs and symptoms of active infection, is acceptable to participate. Serology testing may be performed by an alternative certified laboratory while awaiting central laboratory serology results, if turnaround times delay receipt of test results prior to randomization • Has antibodies to HCV, unless they satisfy 1 of the following conditions: (1) has a history of successful treatment, defined as being negative for HCV RNA ≥ 24 wk after completing antiviral treatment, and has a negative HCV RNA test result at screening, or (2) has a negative HCV RNA test result > 24 wk prior to screening and a negative HCV RNA test result at screening • Has screening laboratory test result of total IgG < 6 g/L • Has screening laboratory test result of albumin < LLN • Has screening laboratory test result of hemoglobin < 80 g/L • Has screening laboratory test result of white blood cell count < 3.0 GI/L • Has screening laboratory test result of absolute neutrophil count < 1.5 GI/L • Has screening laboratory test result of platelet count < 100 GI/L • Has screening laboratory test result of AST ≥2 × ULN (values for ULN should be based on normal reference range for the second trimester in pregnancy) • Has screening laboratory test result of ALT ≥2 × ULN (values for ULN should be based on normal reference range for the second trimester in pregnancy) • Has screening laboratory test result of estimated glomerular filtration rate < 90 mL/min per 1.73 m ^2^ • Has any condition in the current pregnancy (including known genetic defects of the fetus or umbilical cord abnormality) for which, in the opinion of the investigator, participation would not be in the best interest of the participant or fetus/neonate/infant (e.g., compromise the well-being) or that could prevent, limit, or confound the protocol-specified assessments • Has a history of moderate or severe substance or alcohol use disorder according to *Diagnostic and Statistical Manual of Mental Disorders, Fifth Edition* criteria, except nicotine and caffeine, within 1 y prior to screening and during the current pregnancy • History of an unprovoked pulmonary embolism or recurrent deep vein thrombosis

Abbreviations: ALT, alanine aminotransferase; AST, aspartate aminotransferase; BCG, Bacillus Calmette-Guérin; ECG, electrocardiogram; FcRn, neonatal Fc receptor; FNAIT, fetal and neonatal alloimmune thrombocytopenia; GA, gestational age; HBV, hepatitis B virus; HCV, hepatitis C virus; ICF, informed consent form; ICH, intracranial hemorrhage; IgG, immunoglobulin G; IVIG, intravenous immunoglobulin; LLN, lower limit of normal; ULN, upper limit of normal.

Participants will be excluded from the study if they are pregnant with multiple gestations; have a history of severe preeclampsia or severe fetal growth restriction in a previous pregnancy; or have a history of myocardial infarction, unstable ischemic heart disease, stroke, an unprovoked pulmonary embolism, or recurrent deep vein thrombosis. Additional exclusion criteria include a history of recurrent severe or serious infection that required hospitalization or parenteral antibiotics within 8 weeks of screening, severe infection (including opportunistic infection) that required hospitalization or parenteral antibiotics within 8 weeks before screening, COVID-19 infection within 4 weeks prior to the first dose of study intervention, or severe chronic infection. Participants who received corticosteroids, immunosuppression, or other antibody-based therapy for disorders unrelated to the pregnancy, and/or IVIG during the current pregnancy, and participants with known allergies, hypersensitivity, or intolerance to nipocalimab or its excipients are also excluded (see complete exclusion criteria in Table 1 ).

### Study Design

**Fig. 2:**
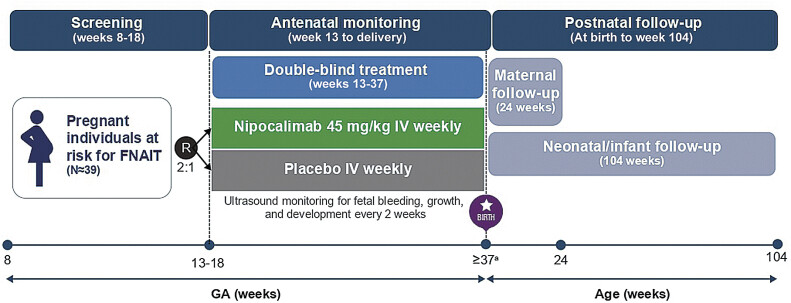
FREESIA-1 study design. FNAIT, fetal and neonatal alloimmune thrombocytopenia; GA, gestational age; IV, intravenous; R, randomization. ^a^ The delivery time and type will occur based on the investigator's judgement. The maternal participant will receive the weekly study intervention until before delivery.

FREESIA-1 is a multicenter, randomized, placebo-controlled, double-blind, phase 3 study that will enroll approximately 39 eligible anti-HPA-1a-alloimmunized pregnant individuals ( Fig. 2 ). Participants are being recruited from maternal-fetal medicine centers at multiple global study sites in Belgium, Brazil, France, Hungary, Israel, Italy, Norway, Slovakia, Slovenia, Spain, Sweden, and Switzerland, with planned sites in Canada and the Czech Republic.

Participants will be randomized 2:1 to receive weekly intravenous nipocalimab or placebo beginning between 13 and 18 weeks GA and continuing until delivery. Participants will be surveilled via fetal ultrasound at baseline and approximately every 2 weeks to assess fetal growth, development, and signs of fetal bleeding. The method of delivery will be determined based on the investigator's judgment, considering the maternal participant's health, the safety of the neonate, and local and national guidelines/practices. If a transfusion is required, HPA-selected platelets will be provided to the neonate if available.

Neonates will undergo a cranial ultrasound scan at birth and prior to hospital discharge to rule out perinatal ICH, undergo blood sampling to monitor platelet counts, and, if needed, receive a platelet transfusion according to the study protocol. Postnatal follow-up periods are 24 weeks for maternal participants and 104 weeks for neonates/infants.

### Study Assessments

A listing of study endpoints is provided in Table 2 . The primary composite endpoint is the adverse outcome of fetal or neonatal death, adjudicated severe bleeding in utero or up to the first week postbirth, or neonatal platelet count at birth < 30 × 10 ^9^ /L. Key secondary efficacy endpoints include adjudicated bleeding in utero up to the first week postbirth in fetuses/neonates and platelet count at birth in neonates. Additional secondary efficacy endpoints include death of a fetus or neonate; platelet count at birth in neonates <10, <30, <50, and <150 × 10 ^9^ /L; nadir platelet count of neonates over the first week postbirth; neonate requiring platelet transfusion(s), including the number of platelet transfusions and number of donor exposures for platelet transfusion(s); adjudicated severe bleeding in utero up to the first week postbirth in fetuses/neonates; and neonate requiring postnatal IVIG for the treatment of thrombocytopenia. The incidence of antibodies to nipocalimab, including neutralizing antibodies, in maternal serum during pregnancy and postpartum is also assessed as a secondary endpoint.

**Table 2:** Study objectives and endpoints

Objectives	Endpoints
Primary	
• To evaluate the superiority of nipocalimab compared with placebo in reducing the risk of severe FNAIT	• Adverse outcome of death or adjudicated severe bleeding in utero up to the first week postbirth, or platelet count at birth < 30 × 10 ^9^ /L in a fetus/neonate
Secondary	
• To evaluate the efficacy of nipocalimab compared with placebo in reducing the risk of FNAIT-related bleeding (key secondary)	• An adjudicated bleeding in utero up to the first week postbirth in a fetus/neonate
• To evaluate the efficacy of nipocalimab compared with placebo in reducing the risk of FNAIT-related thrombocytopenia (key secondary)	• Platelet count at birth in a neonate
• To evaluate the efficacy of nipocalimab compared with placebo in reducing the risk of FNAIT-related outcomes	• Adverse outcome of the death of a fetus/neonate • Platelet count at birth < 10 × 10 ^9^ /L in a neonate • Platelet count at birth < 30 × 10 ^9^ /L in a neonate • Platelet count at birth < 50 × 10 ^9^ /L in a neonate • Platelet count at birth < 150 × 10 ^9^ /L in a neonate • Nadir platelet count of a neonate over the first week postbirth • Neonate requiring platelet transfusion(s), including the number of platelet transfusions and number of donor exposures for platelet transfusion(s) • An adjudicated severe bleeding in utero up to the first week postbirth in a fetus/neonate • A neonate requiring postnatal IVIG for the treatment of thrombocytopenia
• To evaluate the safety of nipocalimab compared with placebo in maternal and neonatal/infant participants	• Maternal participant with a TEAE, SAE, or AESI • Maternal participant with TEAE leading to discontinuation of study intervention • A neonate/infant with a TEAE, SAE, or AESI • A fetus/neonate with a TEAE of bleeding • A neonate with a TEAE of infection • Infant development as measured by the Bayley scales at weeks 52 and 104
• To evaluate the immunogenicity of nipocalimab in maternal participants	• Incidence of antibodies to nipocalimab, including neutralizing antibodies, in maternal serum during pregnancy and postpartum
Exploratory	
• To assess the impact of nipocalimab compared with placebo on pregnancy and neonatal outcomes	• Pregnancy resulting in preterm birth at GA week < 37 • Deliveries performed via cesarean • Pregnancies affected by fetal growth restriction • Neonate with birth weight < 10th percentile of GA
• To evaluate the safety of nipocalimab compared with placebo in maternal and neonatal/infant participants	• Change from baseline in laboratory parameters over time in maternal and neonatal/infant participants (e.g., infant's IgG, response to tetanus vaccines)
• To evaluate the PK of nipocalimab in maternal participants	• Serum nipocalimab concentration in maternal blood over time during pregnancy and postpartum • Nipocalimab concentration in colostrum/breast milk (birth to 4 wk)
• To evaluate the PK of nipocalimab in the neonate	• Nipocalimab concentration in the neonate over time
• To evaluate the PD of nipocalimab in maternal participants and neonates/infants	• Total IgG in maternal and neonate/infant blood over time
• To evaluate the impact of nipocalimab compared with placebo on health-related quality of life and health status in maternal participants and infants	• Maternal health-related quality of life: change from baseline SF-36 v2 acute domains and component scores over time • Maternal health-related quality of life: change from baseline in EQ-5D-5L visual analog scale and EQ-5D-5L index scores over time • Infant health-related quality of life: IQI score over time
• To assess medical resource utilization data in neonates	• Use of intensive care for neonate, measured as length of stay
• To evaluate the effect of nipocalimab compared with placebo on other biomarkers	• Change from baseline in biomarker levels over time (e.g., alloantibodies as exploratory PD)
• To evaluate the effect of nipocalimab compared with placebo on the placental findings	• Histopathologic placenta evaluation • Placenta weight and placenta/birth weight ratio (as a measure of placenta function)

Abbreviations: AESI, adverse event of special interest; EQ-5D-5L, EuroQol Five-Dimension Five-Level Questionnaire; FNAIT, fetal and neonatal alloimmune thrombocytopenia; GA, gestational age; IgG, immunoglobulin G; IQI, Infant Health-Related Quality of Life Instrument; IVIG, intravenous immunoglobulin; PD, pharmacodynamics; PK, pharmacokinetics; SAE, serious adverse event; SF-36 v2, 36-Item Short Form Health Survey Version 2; TEAE, treatment-emergent adverse event.

Safety assessments for both the maternal and neonate/infant participants include monitoring for AEs, serious AEs, and AEs of special interest. Neonatal and infant development is assessed using the Bayley Scales of Infant and Toddler Development, Third Edition, and immune system development is measured using the immunoglobulin profile.

Other selected endpoints include patient- and caregiver-reported outcomes (i.e., 36-Item Short Form Health Survey Version 2 acute scores over time, EuroQol Five-Dimension Five-Level Questionnaire scores over time, and Infant Health-Related Quality of Life Instrument scores over time), as well as pharmacokinetics and pharmacodynamics of nipocalimab.

### Statistical Analyses

A planned sample size of 39 participants (26 on nipocalimab and 13 on placebo) was estimated to provide the trial with > 90% power to detect a difference for the primary endpoint, assuming the proportion of participants achieving the primary endpoint was 8 and 60% in the nipocalimab and placebo groups, respectively, based on a Cochran–Mantel–Haenszel (CMH) test at a two-sided significance level of 5%. The 8% achievement of the primary endpoint in the nipocalimab group was determined based on published literature, where 12% of IVIG-treated standard-risk FNAIT pregnancies achieved a birth platelet count < 30 × 10 ^9^ /L, 24 25 26 applying a relative risk reduction of one-third for nipocalimab compared with IVIG. 20 The 60% achievement in the placebo group was determined based on a historical Norwegian study, where 60% of untreated standard-risk participants had a birth platelet count < 30 × 10 ^9^ /L. 27 The primary endpoint will be analyzed using a CMH test, stratified by region (i.e., non-Nordic or Nordic country) at a two-sided significance level of 5%. The treatment group comparisons for the key secondary endpoint of the adjudicated bleeding endpoint will be performed using the stratified CMH test and for platelet counts using the stratified van Elteren test.

A multiplicity adjustment testing procedure will be used to control the type 1 family-wise error rate at a two-sided significance level of 5% for the primary and key secondary endpoints. The testing continues sequentially from the primary endpoint until the first instance where the null hypothesis is not rejected. No further formal hypothesis testing will be conducted. *p* -Values for other endpoints will be considered nominal.

## Discussion

FNAIT is a rare and potentially life-threatening alloimmune condition associated with morbidity and mortality in fetuses and newborns. 18 Current treatment options for pregnancies at risk of FNAIT are limited and result in inconsistent clinical outcomes. Moreover, there are no approved preventive treatments for FNAIT in at-risk pregnancies. 2 19 Therefore, preventive treatments for FNAIT remain a substantial unmet need. Evidence from other alloantibody-mediated perinatal diseases suggests that nipocalimab may have utility as a potential therapy in treating FNAIT. 20 21 28 29 FREESIA-1 is the first placebo-controlled, randomized, multicenter clinical trial investigating the safety and efficacy of nipocalimab in alloimmunized pregnant individuals in the standard risk group. Nipocalimab may reduce the risk and severity of FNAIT through blockade of IgG binding to FcRn in the blood and placenta, thereby lowering maternal concentrations of IgG (including pathogenic alloantibodies) available for transfer and blocking maternal transfer of IgG (including pathogenic alloantibodies) to the fetal circulation.

FREESIA-1 focuses on standard-risk pregnancies, as this population has a lower risk for ICH, and IVIG treatment practices are not standardized for these pregnancies due to an unclear benefit-risk profile. 10 Depending on the country, management of a standard-risk FNAIT pregnancy ranges from no treatment to different doses of IVIG. 10 19 For example, in some European countries, like Norway, Sweden, and the Netherlands, IVIG is not always recommended in standard-risk pregnancies. 19 This variability in clinical practice allows for a randomized, placebo-controlled study design. In contrast, individuals in the high-risk group are generally advised to receive antenatal IVIG treatment due to the substantially increased risk of recurrent ICH in subsequent pregnancies and the observed risk reduction when using IVIG for this group. 2 19

Study endpoints, including both safety and efficacy assessments of nipocalimab, are consistent with the standard-of-care management of alloimmunized pregnant individuals in the standard risk group to provide clinically meaningful information. The primary composite endpoint encompasses typical clinical outcomes relevant to patients with FNAIT, including adverse outcomes of death or adjudicated severe bleeding in utero up to the first week postbirth or platelet count at birth < 30 × 10 ^9^ /L in a fetus/neonate.

As nipocalimab reduces maternal serum IgG levels and placental transfer of both pathogenic and beneficial IgG antibodies, antenatal treatment with nipocalimab may affect maternal immune functions and impair neonatal passive immunity, as well as cause a potential risk of both maternal and neonatal infections. 20 In this study, several strategies are employed to mitigate this infection risk, such as excluding maternal participants with a history of serious infection, solid organ or bone marrow transplantation, or immunodeficiency syndrome. Additionally, maternal participants and their neonates/infants will be closely monitored for any signs or symptoms of infection. However, based on phase 1 to 3 studies in nonpregnant and pregnant participants conducted to date, nipocalimab demonstrated an acceptable safety profile without unusual infections, broad immunosuppression, or impact on vaccine responses. 20 21 28 29 30 31 32 33 34 Therefore, nipocalimab is hypothesized to have an acceptable safety profile in standard-risk pregnancies for FNAIT based on the available safety and toxicology findings.

The multicenter study design of FREESIA-1 supports the generalizability of the results to pregnancies at risk of FNAIT in clinical settings. Given the rarity of the disease, the study is adequately powered with an appropriate sample size to detect a difference in the primary endpoint between nipocalimab and placebo. Additionally, the 2:1 randomization ratio ensures that more participants receive nipocalimab compared with placebo, which expands the safety dataset for this indication. However, the 2:1 randomization may also present a limitation, as it may hinder data interpretation, such as the ability to identify drug-related AEs at a low frequency. To complement the findings from the placebo-controlled group in FREESIA-1, additional data will be gathered in an ongoing global, multicenter, retrospective chart review study, which is characterizing the treatment patterns, outcomes, and management of pregnancies at risk of FNAIT. Finally, this study is not powered to determine whether nipocalimab prevents ICH given the very low risk of ICH in standard-risk pregnancies.

## Conclusion

The FREESIA-1 study is the first multicenter, randomized, placebo-controlled clinical trial designed to evaluate the safety and efficacy of nipocalimab as a potential preventive and noninvasive intervention for the treatment of alloimmunized pregnant individuals in the standard risk group. The outcomes of this study will contribute to further developing nipocalimab as a potential pharmacologic treatment option for reducing the risk of severe FNAIT, a rare disease with significant unmet medical need associated with significant morbidity and mortality for the fetus/neonate.
